# Impact of a factor Xa inhibitor (apixaban) on SIV pathogenesis and response to antiretroviral therapy

**DOI:** 10.1172/jci.insight.202434

**Published:** 2026-04-08

**Authors:** Cuiling Xu, Haritha Annapureddy, Lilly Carson, Vansh Khurana, Ranjit Sivanandham, Sindhuja Sivanandham, Tianyu He, Kevin D. Raehtz, Janet Kim, Christie Biber, Norma Arbujas-Silva, Mohammed Daira, Sudhapriya Kandasamy, Matthew J. Feinstein, Irini Sereti, Cristian Apetrei, Ivona Pandrea

**Affiliations:** 1 Department of Pathology and; 2 Division of Infectious Diseases, Department of Medicine, School of Medicine, University of Pittsburgh, Pittsburgh, Pennsylvania, USA.; 3 Department of Pathology and; 4 Division of Cardiology, Department of Medicine, Feinberg School of Medicine, Northwestern University, Chicago, Illinois, USA.; 5 Division of Intramural Research, National Institute of Allergy and Infectious Diseases, National Institutes of Health (NIH), Bethesda, Maryland, USA.; 6 Department of Infectious Diseases and Microbiology, Graduate School of Public Health, University of Pittsburgh, Pittsburgh, Pennsylvania, USA.

**Keywords:** AIDS/HIV, Infectious disease, Virology, AIDS vaccine, Coagulation

## Abstract

Antiretroviral therapy (ART) has prolonged the life expectancy of persons living with HIV, the majority of whom are now older than 50 years. Aging people with HIV are at increased risk for cardiovascular events driven by HIV-related inflammation and hypercoagulation. Apixaban is a factor Xa inhibitor that reduces cardiovascular risks and treats stroke, deep vein thrombosis, and pulmonary embolism. We assessed apixaban’s impact on key parameters of HIV/SIV pathogenesis in SIV-infected, aged rhesus macaques (RMs) receiving ART. Inflammation, coagulation, T cell subsets, B cells, and macrophages and their immune activation status were monitored throughout the study. We found no significant differences between the apixaban-treated and control groups for virus replication or CD4^+^ T cell recovery in blood and tissues after ART. Apixaban did not significantly affect D-dimer, immune activation, or inflammation of SIV-infected, ART-treated RMs. Apixaban-treated RMs experienced multiple bleeding episodes, tissue hemorrhages, and myocardial infarctions, as demonstrated by pathological examination of necropsy-collected tissues. Given apixaban’s lack of effect on immune activation, CD4^+^ T cell restoration, and inflammation, along with increased risk of hemorrhage, factor Xa inhibition may not be an efficient or safe option to target and prevent cardiovascular events in aging people with HIV.

## Abstract 2

Addition of a factor Xa inhibitor (Apixaban) to ART has no discernible impact on the pathogenesis of SIV infection and does not reduce hypercoagulability and inflammation

## Introduction

A hypercoagulable state with increased D-dimer and thrombin-antithrombin levels has been consistently observed in persons living with HIV and in SIV-infected nonhuman primates (NHPs) and is a strong independent predictor of mortality (1–4). Abnormal levels of endothelial and platelet activation markers have also been documented in people living with HIV (5, 6). In animal models of HIV infection, hypercoagulation is specifically associated with pathogenic infections but is absent in natural hosts of SIV, and it appears to be driven not by the virus itself but by increased levels of chronic inflammation (3). In both humans and NHPs, hypercoagulability related to HIV/SIV has been associated with cardiovascular lesions and other comorbidities and accelerated aging (7, 8). There is a strong association between HIV infection and thrombotic microangiopathy. In the era before antiretroviral therapy (ART), the incidence of thrombotic microangiopathy was as high as 7% (9), and up to 20% of people diagnosed with thrombotic microangiopathy were HIV-positive. Survival after thrombotic microangiopathy diagnosis is poor (10, 11), irrespective of treatment initiation. Thrombotic events are 10 times more frequent in people living with HIV (12, 13) compared with the general population (14, 15). Our previous studies demonstrated that increased D-dimer/thrombin-antithrombin levels are associated with thrombotic microangiopathy lesions in multiple tissues in untreated NHPs with SIV (3). Although only a few human autopsy studies have been performed, they also revealed high rates of previously undiagnosed thromboembolism among patients with AIDS (16).

Elderly individuals are disproportionally affected by a prothrombotic status associated with an increased risk of thromboembolism, stroke, and myocardial infarction, even in the absence of HIV infection (17–23). We also previously determined that, similar to humans, NHPs experience age-related increases in coagulation markers (24). It is therefore conceivable that the consequences of HIV-related hypercoagulability will be particularly severe in elderly patients and may explain the higher frequency of cardiovascular events in this age group (25, 26).

In addition to its critical contribution to cardiovascular comorbidities, hypercoagulation may negatively affect responses to ART in older people living with HIV (27). Coagulation factors may promote immune activation and inflammation, which result in excessive CD4^+^ T cell death (28). Coagulation is also closely intertwined with fibrosis: thrombin activation of PAR-1 and PAR-2 promotes fibroblast proliferation and collagen deposition either directly or via induction of host mediators, such as platelet-derived growth factor or connective tissue growth factor (29–33). This may link coagulation to a poor response to ART, by impeding immune restoration in individuals with HIV and in SIV-infected NHPs (34). These observations suggest that hypercoagulation may be one of the key elements involved in the frequent immunological failures observed in elderly patients (27).

With the advent of effective ART, which provides robust and sustained suppression of the virus, the prognosis of HIV infection has significantly improved. However, ART is not fully successful in abrogating coagulation abnormalities (35). If an individual recently infected with HIV starts ART, their prognosis is 59 years, which is close to the normal lifespan (36). Consequently, the majority of people living with HIV in the United States are now older than 50 years (37). Furthermore, more than 50% of people with HIV are over the age of 50 in low- and middle-income countries, and it is projected that more than 70% of people with HIV worldwide will be over the age of 50 by 2030 (38). Higher age is also associated with a hypercoagulable state even in the absence of HIV infection (39); therefore, assessing whether control of hypercoagulation in older populations could alleviate comorbidities and improve quality of life and life expectancy of individuals with HIV represents a critical and unmet objective of AIDS research (7).

We have previously shown that tissue factor inhibition with an experimental drug alleviates hypercoagulation and partially improves immune activation in young SIV-infected NHPs (40). FDA-approved tissue-factor inhibitors are not yet available on the market as a treatment option. Therefore, new strategies to normalize coagulation in patients with HIV using FDA-approved drugs need to be investigated to fill this gap. Testing anticoagulants that act at different levels of the coagulation cascade could enable us to dissect the mechanisms of hypercoagulation in individuals with HIV and open new avenues for adjunct therapies aimed at controlling residual inflammation. It is also important to examine whether targeting the coagulation cascade at different levels will have the same effect as previous studies targeting tissue factor, which initiates coagulation. However, performing these treatments in patients with HIV carries intrinsic risks because anticoagulants may predispose patients to hemorrhagic events and toxic outcomes due to drug-drug interactions with ART; animal models can be used instead to achieve this goal.

Several commercially available anticoagulants are designed to inhibit Factor Xa, which is located at the top of the common coagulation pathway and directly below factor X. Tackling factor Xa has the potential to inhibit both coagulation pathways and reduce inflammation. However, a recent randomized clinical trial that employed the factor Xa inhibitor edoxaban reported that, while the drug reduced coagulation activity, it did not affect inflammation or immune activation in individuals with HIV (41). Here, we employed a different factor Xa inhibitor, apixaban, which is an FDA-approved drug used to reduce the risk of stroke and blood clots and treat deep vein thrombosis and pulmonary embolism (42–44). Of all Xa inhibitors available, apixaban has the best toxicity profile and the lowest risk of drug-drug interactions with ART in patients with HIV (41, 45). The latter is an important drug selection criterion, as potential side effects and pharmacokinetic interactions between anticoagulants and antiretrovirals could limit their use in people with HIV. A recent study reported that apixaban induces fewer major hemorrhagic events in ART-treated older adults with HIV and atrial fibrillation compared with other anticoagulants, such as warfarin and rivaroxaban (46). Although this study was published after we initiated our NHP trial, it supports our choice of apixaban and its usage in our study design. The animal model also gave us the advantage of having access to tissues of the subjects treated with apixaban, thus allowing us to take a further step in assessing its safety and efficacy in SIV/ART settings. We were also able to assess the efficacy of anticoagulation in affecting major parameters of SIV infection and thus determine whether or not the benefits of apixaban are offsetting its risks.

Previous studies that have been conducted to test the usefulness of anticoagulants in HIV infection as a method of preventing comorbidities in patients (40, 41, 47, 48) did not have positive outcomes; it was therefore important to find the most appropriate pathway that can be safely targeted in aging individuals with HIV.

Our goals were the following: (a) to assess apixaban’s efficacy in reducing hypercoagulation in animals infected with SIV; (b) to determine the side effects and toxicity of apixaban combined with ART with a focus on the tissues collected from the treated NHPs; (c) to investigate apixaban’s effect on immune activation and inflammation responses and virus suppression while on ART; and (d) to examine apixaban’s impact on key biomarkers of SIV pathogenesis (i.e., CD4^+^ T cell recovery or viral loads). We report that apixaban did not significantly affect immune activation, inflammation, or coagulation status in SIV-infected ART-treated rhesus macaques (RMs) but did cause bleeding events during the follow-up period and tissue hemorrhage, thus raising concerns on whether such therapy would be safe for people living with HIV.

## Results

### Study design

**Figure 1:**
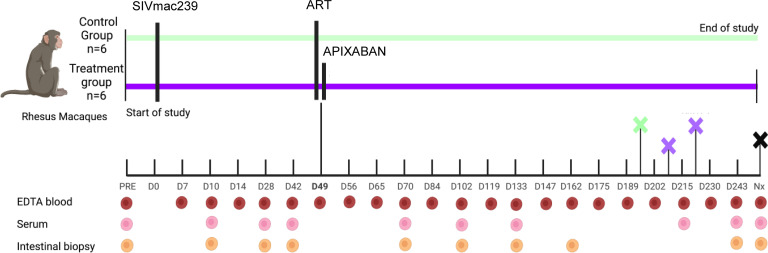
Study design. Animal groups, sampling schedules, and sample types are shown.

The study group comprised 6 female RMs (*Macaca mulatta*) aged 17–19 years. A control group of 6 female RMs aged 17–21 years was also included and subjected to all the procedures performed in the study group, except the apixaban administration (Figure 1). Anticoagulant treatment was given to older animals to model aging patients with HIV, who are the most affected by hypercoagulation and cardiovascular comorbidities. All macaques were i.v.-infected with 100 tissue culture infectious doses (TCID_50_) of SIVmac239 (49) and were closely monitored throughout the follow-up period. Coformulated ART (50) was initiated in all RMs at 49 days after infection (dpi) for the remainder of the follow-up period, as described (51). Apixaban (2.5 mg, orally, twice a day) was also initiated concomitantly with ART for the RMs in the study group to assess its impact on key parameters of SIV infection (survival, viral replication, CD4^+^ T cell counts and activation status, systemic inflammation, and coagulation status). To avoid hemorrhages, apixaban was discontinued 2 days prior to intestinal and lymph node biopsies and resumed 1 day after the procedures.

### Apixaban led to adverse events in the treatment group

#### Clinical and biological follow-up.

**Figure 2:**
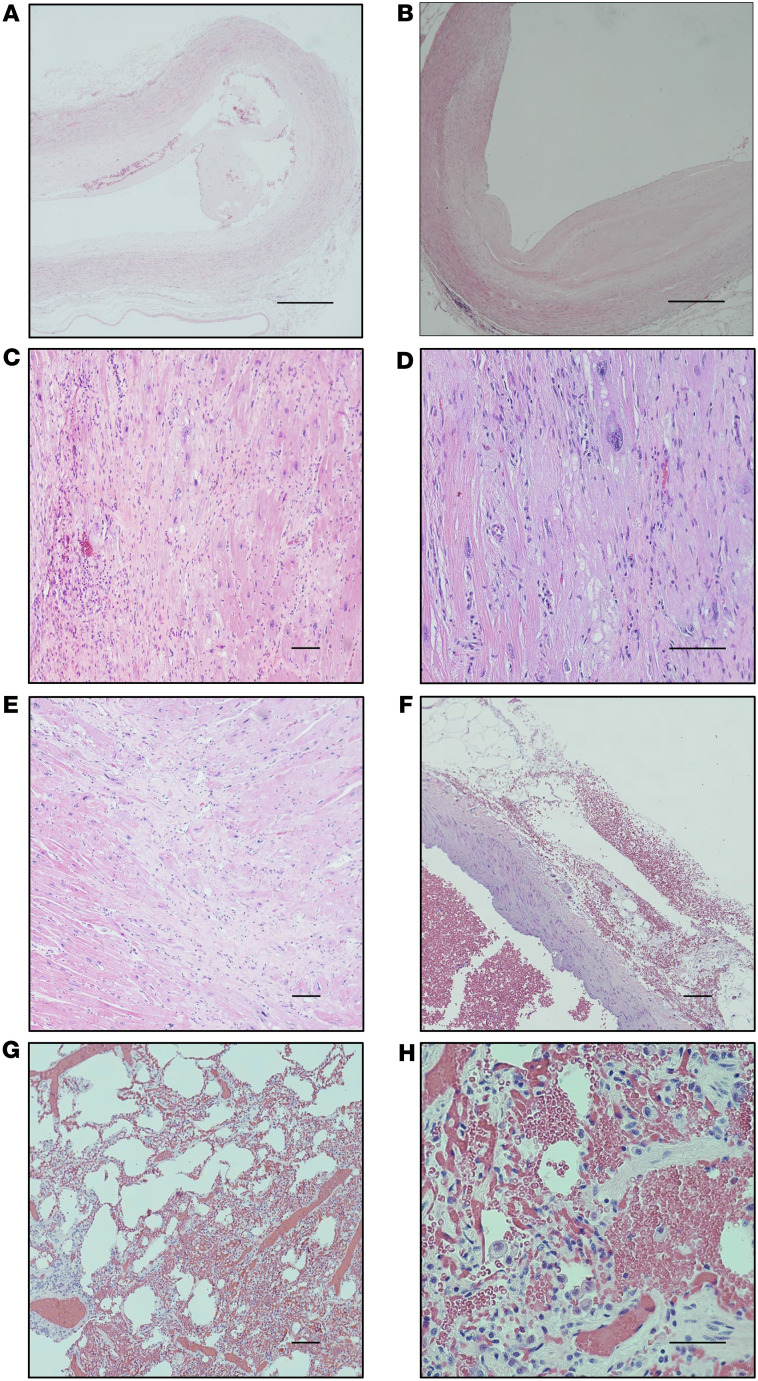
Pathological findings in apixaban-treated rhesus macaques. One RM experienced an aortic dissection characterized by the tearing of the aorta intima and the dissection of the blood underneath intima. Dissection occurred at a site of an atherosclerotic plaque (**A**). Several RMs had uncomplicated atherosclerotic plaques located in the abdominal aorta (**B**). One RM experienced a recent myocardial infarction, as shown by the increased number of immune cells infiltrating the area with necrotic myocardial fibers (**C**) and the hypertrophic myocardial fibers (large, irregular nuclei) in an attempt to compensate for the loss of necrotic myocardial fibers at the periphery at the infarction area (**D**). Old myocardial scars were present in several RMs and were characterized by more mature collagen tissue and a paucity of immune cells (**E**). Small hemorrhages were present in the adipose tissue of the epicardium (**F**) and in the lung (**G**). A detailed image (**F**) shows erythrocytes in the alveolar lumen. Scale bars: 500 μm (**A** and **B**), 100 μm (**C**–**G**), 50 μm (**H**). Magnifications: 4× (**A** and **B**); 10× (**C**, **E**, and **G**); 20× (**D** and **F**); 40× (**H**).

All 6 apixaban-treated RMs experienced significant bruising throughout the follow-up period and between 3 and 4 episodes of hematuria. Additionally, 4 animals experienced intermittent bleeding gums. These bleeding episodes led to an early study interruption, with multiple RMs receiving apixaban being euthanized starting at 206 dpi, and study suspension before 1 year after infection. A complete histological examination was performed using tissue sections stained with H&E; the lesions are summarized in Supplemental Table 1, and their quantifications are in Supplemental Table 2 (supplemental material available online with this article; https://doi.org/10.1172/jci.insight.202434DS1). Briefly, 1 RM that was euthanized early had an aortic dissection developed on an atherosclerotic background. Hemorrhages were present underneath the thoracic and abdominal aorta intima, which was detached from the aortic wall (Figure 2A). Atherosclerotic lesions were found in the abdominal aorta of all RMs (Figure 2B), but their severity was variable. None of the atherosclerotic plaques obstructed more than 50% of the blood vessel lumen, which would have posed a high risk for acute cardiovascular events. However, heavy mononuclear infiltration observed in a myocardial scar (Figure 2, C and D) suggests a recent myocardial infarction in that animal. Another animal had myocardial scars indicative of old infarctions (Figure 2E). However, similar lesions were also present in controls (Supplemental Table 1), suggesting that they were most likely related to the age of the animals rather than any other risk factor. Small hemorrhages were also present in the pericardium in 2 RMs (Figure 2F) and in the lung, in 3 RMs (Figure 2, G and H). Fatty infiltration in the heart and lymph nodes was observed in several RMs, both in the apixaban group and in the controls (Supplemental Table 1), suggesting that these features are associated with old age and possibly SIV infection and ART. Lymphoid depletion occurred in both controls and the apixaban group, being slightly more prominent in the latter (Supplemental Table 1). Only 1 control was euthanized during the follow-up period (194 dpi) because of clinical complications unrelated to SIV infection or treatment. Remaining controls were followed up to 1 year after infection.

#### Laboratory assessment.

**Figure 3:**
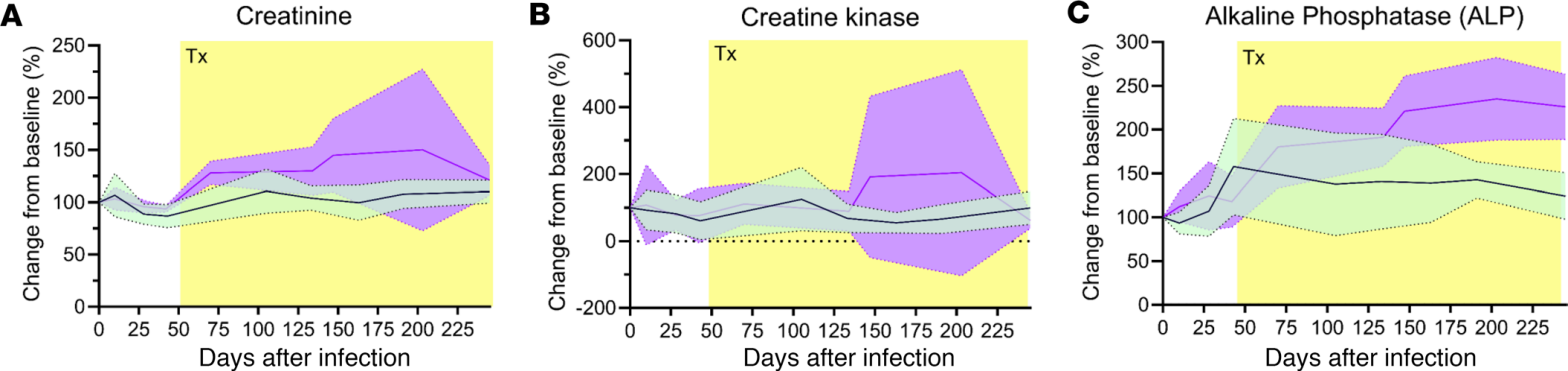
Apixaban administration has minimal laboratory toxicity in SIVmac-infected rhesus macaques receiving ART. The levels of creatinine (**A**) and creatinine kinase (**B**) were similar between the apixaban-treated RMs and controls. During the follow-up period, the levels of alkaline phosphatase increased in the apixaban-treated group to twice the levels observed in controls (**C**), yet without reaching statistical significance. Apixaban-treated RMs are depicted in violet; controls are depicted in light green. Shown are the average of the changes from the baseline levels (%) and the ranges of the SEMs. Tx refers to both antiretrovirals and apixaban in the treated RMs.

Chemistry panels were used to assess the potential toxicity of apixaban and ART. Increased levels of creatinine, a common marker used to measure kidney function, were observed in the apixaban group compared with controls (Figure 3A). These differences were not significant, and only 1 RM from the apixaban group exceeded the normal range of creatinine values of elderly RMs (not shown). The apixaban group also displayed increased levels of creatine kinase, which may be indicative of skeletal muscle, heart, or brain damage or degeneration (Figure 3B). Again, none of these exceeded the normal range of values for RMs of this age, and the increases did not immediately follow apixaban initiation. Alkaline phosphatase (ALP), a common marker of liver damage, displayed similar levels for both groups prior to treatment. ALP levels increased in both groups after ART and apixaban initiation (Figure 3C), and they continued to increase and remained significantly elevated until the end of the study in the apixaban group but without exceeding the normal range of values for old RMs. Thus, although chemistry testing did not indicate overt toxicity in the RMs treated with apixaban and ART, the clear upward trend compared with controls may indicate relative toxicity of the drug.

### Apixaban does not impact virus replication

**Figure 4:**
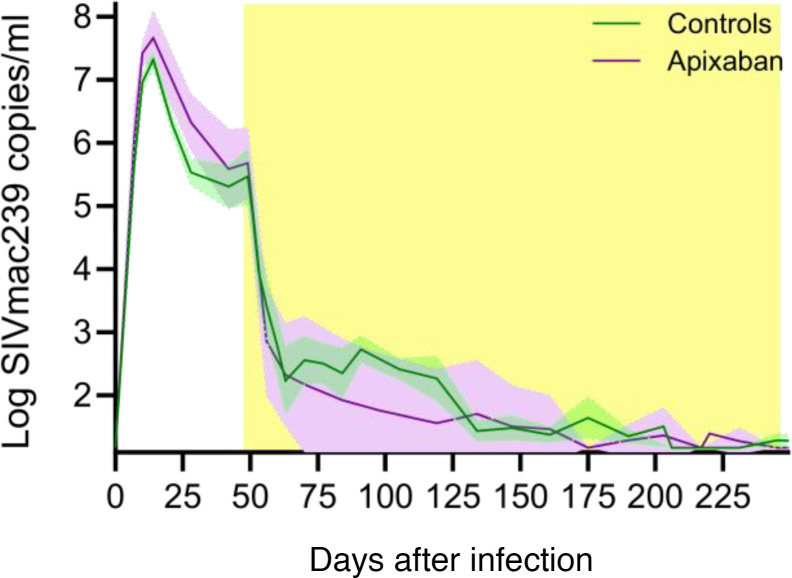
Apixaban administration does not affect virus replication in SIVmac-infected rhesus macaques receiving ART. Plasma viral loads were not different between the apixaban-treated RMs and controls. Apixaban-treated RMs are depicted in violet; controls are depicted in light green. Shown are the average viral loads (log of the SIVmac239 RNA copies/mL of plasma) and the ranges of the SEMs. Tx refers to both antiretrovirals and apixaban in the treated RMs.

Plasma viral loads were similar in the apixaban-treated and control RMs, suggesting no significant impact of the anticoagulant treatment on viral replication (Figure 4).

### Apixaban has no discernible effect on the recovery and activation status of circulating CD4+ T cells

**Figure 5:**
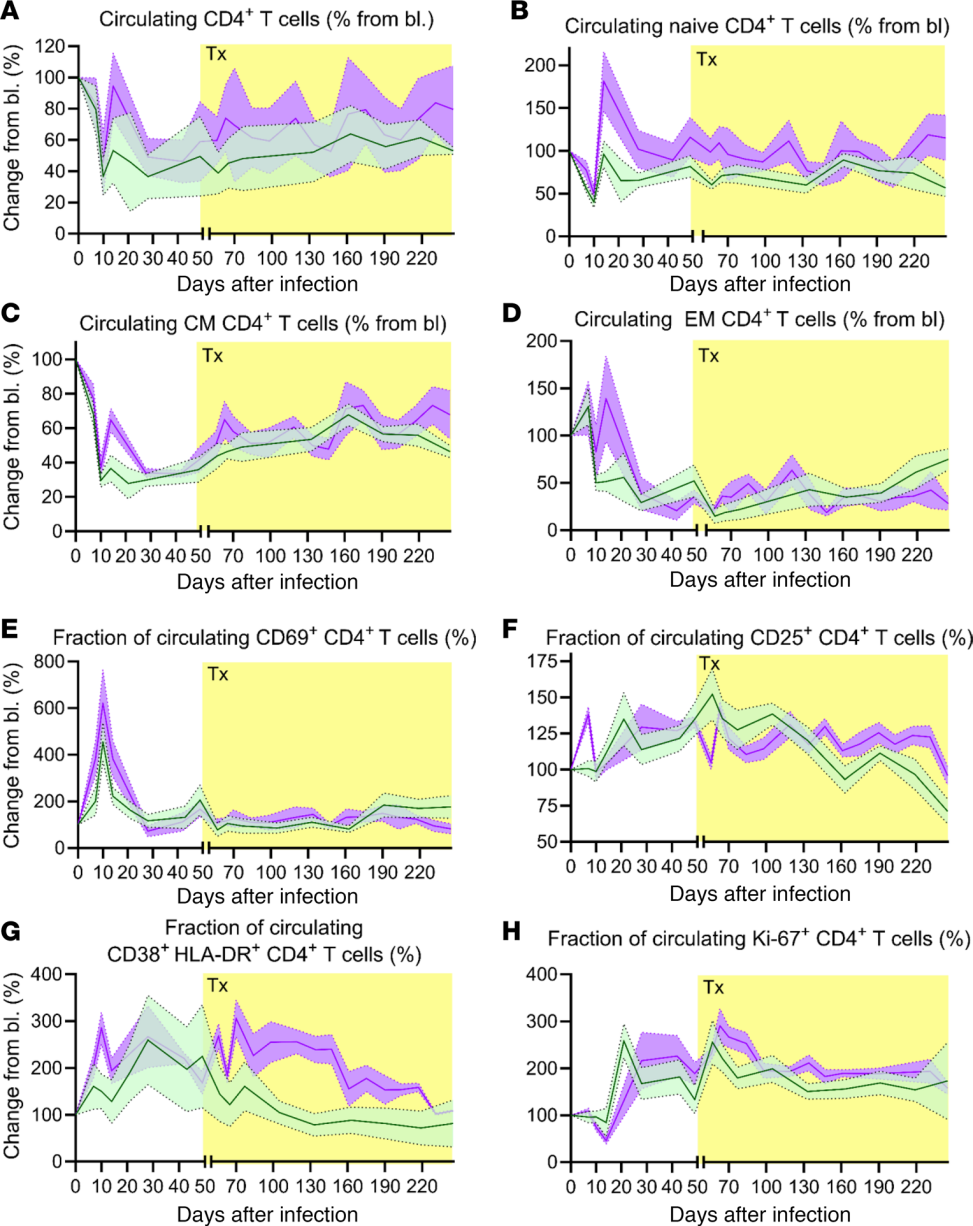
Apixaban administration does not alter the dynamics of circulating CD4^+^ T cells in SIVmac-infected rhesus macaques receiving ART. The magnitude and timing of the CD4^+^ T cell changes were similar in the 2 groups with regard to total circulating CD4^+^ T cells (**A**), as well as the memory subtypes: naive (**B**), central memory (**C**), and effector memory (**D**). Peripheral CD4^+^ T cell activation and proliferation were not different between the 2 groups, as illustrated by the dynamics of the CD4^+^ T cells expressing CD69 (**E**), CD25 (**F**), CD38 and HLA-DR (**G**), and Ki-67 (**H**). All the changes in these populations are illustrated as change from the baseline level (bl; %). Apixaban-treated RMs are depicted in violet; controls are depicted in light green. Data shown as mean ± SEM. Tx refers to both antiretrovirals and apixaban in the treated group.

To assess the effect of apixaban on circulating CD4^+^ T cells, we monitored the recovery and activation status of CD4^+^ T cells through flow cytometry (Supplemental Figure 1 and Figure 5). For a more accurate assessment, we compared CD4^+^ T cell counts at each time point with an average of the baseline values collected prior to infection for each RM. This allowed us to follow the trends of both apixaban-treated and control RMs while factoring in potential differences between the RMs prior to infection and treatment.

After an initial depletion upon the SIV infection, which was similar in both groups, the initial recovery of the total (Figure 5A), naive (Figure 5B), and central memory (Figure 5C) circulating CD4^+^ T cells was more prominent, yet still very transient, in the apixaban-treated RMs than in controls. However, these differences occurred before the initiation of either ART and apixaban and did not reach significance. By the time of apixaban initiation, CD4^+^ T cell levels were similar between the 2 groups, and they did not substantially change with treatment. At the end of the follow-up period, total (Figure 5A), naive (Figure 5B), and central memory (Figure 5C) CD4^+^ T cells were slightly higher in the apixaban-treated RMs than in controls, without reaching statistical significance. CD4^+^ effector memory T cells transiently increased after infection in both groups before steeply declining after the peak of viral replication (Figure 5D), with no significant differences between the 2 groups. Because the differences between groups were minimal and did not reach statistical significance, we concluded that apixaban had no effect on the recovery of circulating CD4^+^ T cells and their subsets.

We next evaluated apixaban’s effect on the activation status of circulating CD4^+^ T cells. There were no significant differences between the groups for CD69^+^ (Figure 5E), CD25^+^ (Figure 5F), or Ki-67^+^ CD4^+^ T cells (Figure 5H) either before or after the initiation of apixaban and ART. HLA-DR^+^ CD38^+^ CD4^+^ T cell levels were similar between groups prior to treatment and remained increased for a longer period of time in the apixaban-treated RMs (Figure 5G); then, they gradually decreased so that at the completion of the study, the fractions of CD4^+^ T cells expressing HLA-DR and CD38 were similar in the 2 groups. As such, we concluded that apixaban had little to no effect on the activation status of circulating CD4^+^ T cells.

### Circulating CD8+ T cells and their subsets were higher in the apixaban-treated RMs independent of anticoagulant administration

**Figure 6:**
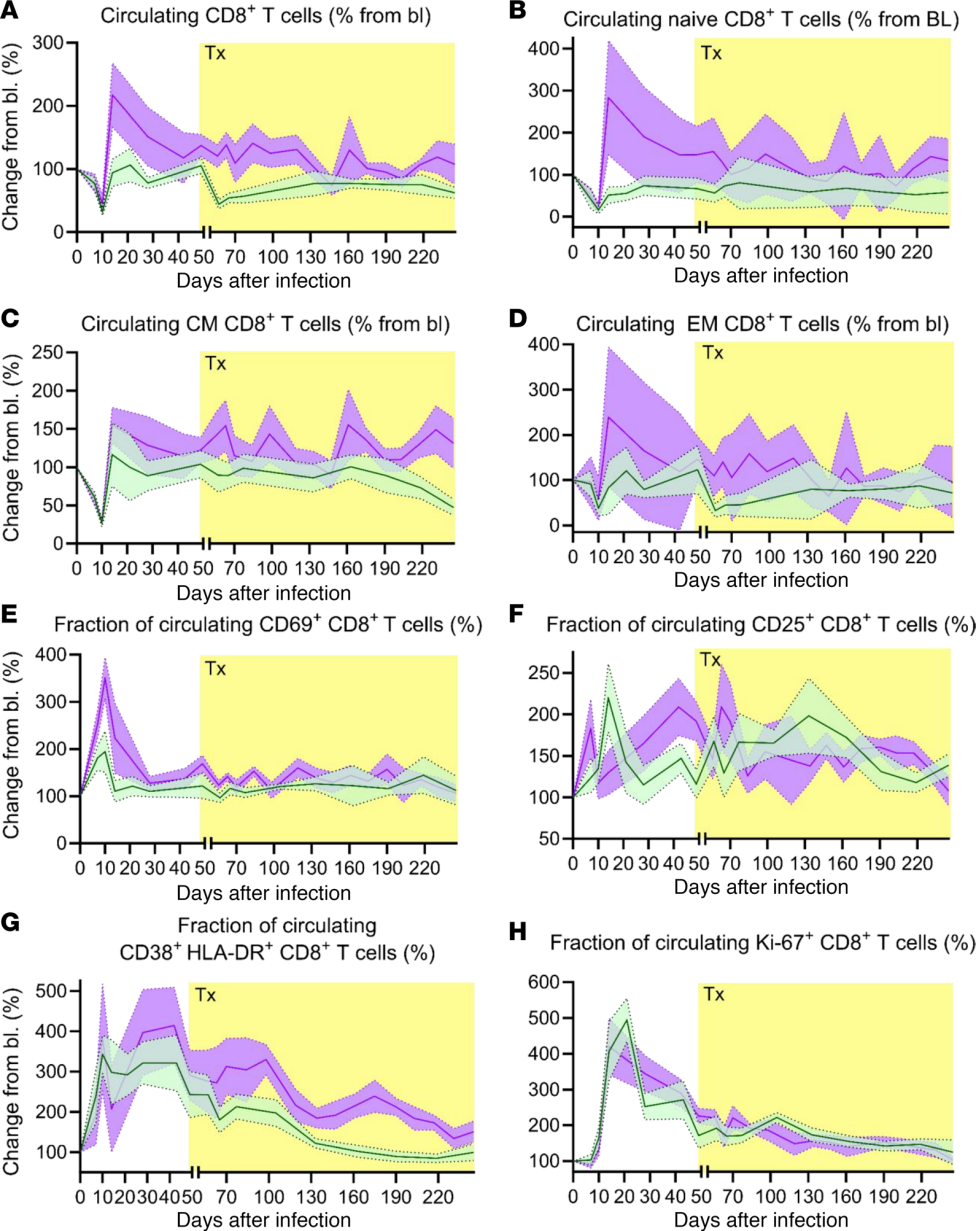
Apixaban administration does not alter the dynamics of circulating CD8^+^ T cells in SIVmac-infected rhesus macaques receiving ART. The dynamics of CD8^+^ T cells were similar in the 2 groups with regard to total circulating CD8^+^ T cells (**A**), as well as the memory subtypes: naive (**B**), central memory (**C**), and effector memory (**D**). Peripheral CD8^+^ T cell activation and proliferation were not different between the 2 groups, as illustrated by the dynamics of CD8^+^ T cells expressing CD69 (**E**), CD25 (**F**), CD38 and HLA-DR (**G**), and Ki-67 (**H**). All the changes in these populations are illustrated as change from the baseline level(bl; %). Apixaban-treated RMs are depicted in violet; controls are depicted in light green. Data are shown as mean ± SEM. Tx refers to both antiretrovirals and apixaban in the treated group.

Flow cytometric assessment (Supplemental Figure 1) showed that, upon SIV infection, total CD8^+^ T cells (Figure 6A), along with naive (Figure 6B), central memory (Figure 6C), and effector memory (Figure 6D) subsets of CD8^+^ T cells decreased similarly in both groups. Their early recovery was more robust in the apixaban group, and they remained higher throughout the follow-up period (Figure 6, A–D). We thus concluded that total CD8^+^ T cells and their memory subsets were higher in the apixaban-treated RMs independent of anticoagulant administration.

Furthermore, there were minimal differences between groups with regard to the expression of immune activation and proliferation markers by CD8^+^ T cells. The frequency of CD69^+^CD8^+^ T cells was higher in the apixaban-treated RMs during acute infection, but reached similar levels in both groups very soon after the initiation of treatment and throughout the follow-up period (Figure 6E). CD25^+^CD8^+^ T cells were increased in controls during acute infection and at the conclusion of the study, but were slightly higher in the treatment group immediately prior to and after the initiation of treatment (Figure 6F). None of these differences reached significance. HLA-DR^+^CD38^+^CD8^+^ T cells were higher in the apixaban group than in controls (Figure 6G). Conversely, the fraction of Ki-67^+^-expressing CD8^+^ T cells was higher in controls during acute infection, reached similar levels in both groups prior to treatment, and remained increased throughout the follow-up period (Figure 6H). Thus, our results indicate that apixaban had no effect on the activation and proliferation status of the circulating CD8^+^ T cells.

### Apixaban has no effect on CD4+ T cell recovery and cell proliferation in the gut

**Figure 7:**
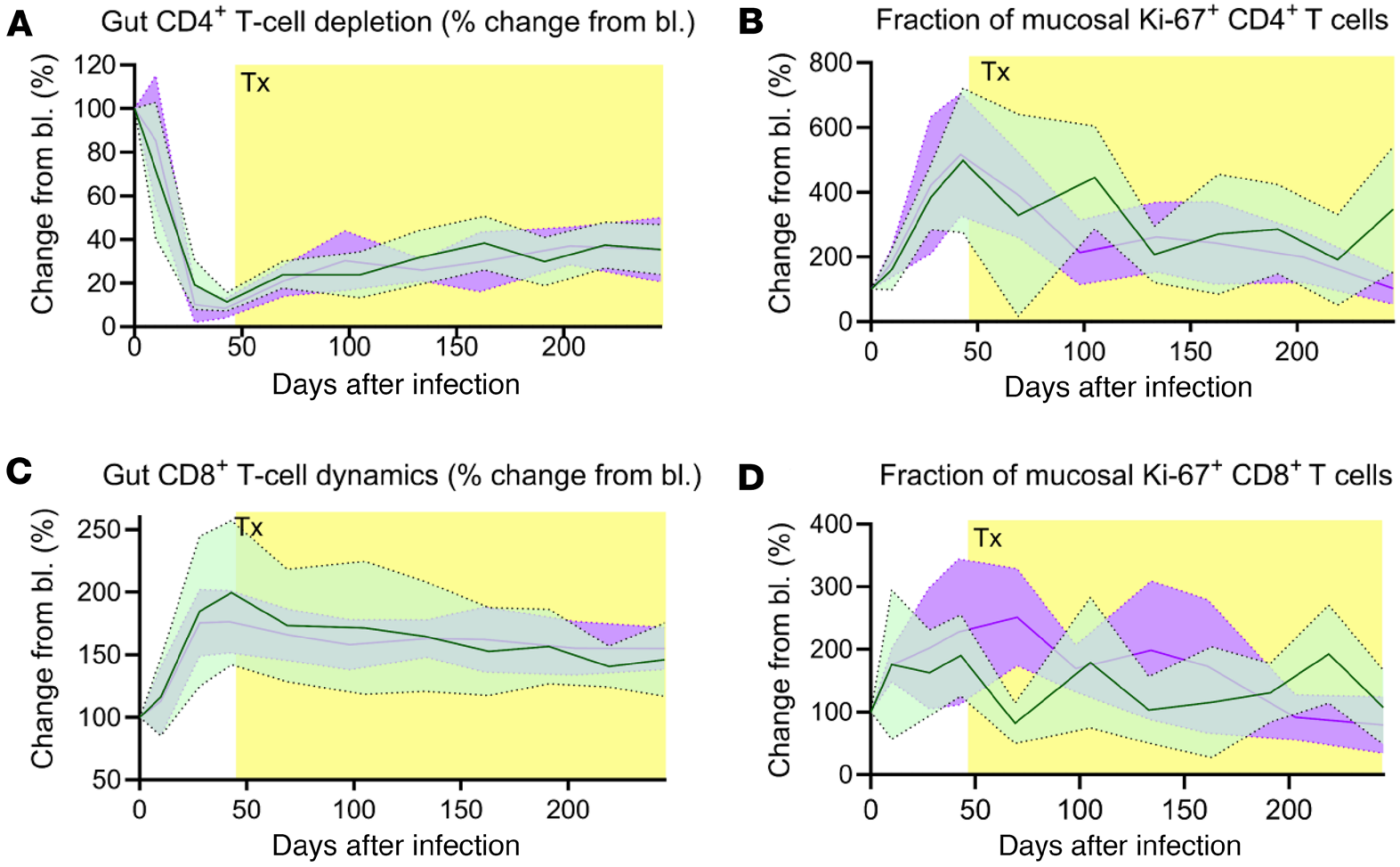
Apixaban administration does not alter the dynamics of mucosal T cells and their proliferation status in SIVmac-infected rhesus macaques receiving ART. The dynamics of the mucosal CD4^+^ (**A**) and CD8^+^ T cells (**C**) were similar in the 2 groups, as were their proliferating fractions: Ki-67^+^ CD4^+^ T cells (**B**) and Ki-67^+^ CD8^+^ T cells (**D**). All the changes in these populations are illustrated as change from the baseline level (bl; %). Apixaban-treated RMs are depicted in violet; controls are depicted in light green. Data are shown as mean ± SEM. Tx refers to both antiretrovirals and apixaban in the treated group.

Flow cytometric assessment showed that, following infection, both apixaban and control groups displayed significant depletion of total CD4^+^ T cells in the gut, and similar slow recovery from depletion after the initiation of ART, with no significant differences between the 2 groups during the follow-up period (Figure 7A). Ki-67^+^ CD4^+^ T cell levels drastically increased in the gut after infection and acute CD4^+^ T cell depletion (Figure 7B). After ART initiation, mucosal Ki-67^+^ CD4^+^ T cells decreased in both groups. The control group yielded slightly higher levels of Ki-67^+^ CD4^+^ T cells than the apixaban-treated RMs. Mucosal CD8^+^ T cells increased after infection and slowly returned to the baseline levels after the initiation of ART (Figure 7C); their dynamics being similar in the 2 groups. Mucosal Ki-67^+^ CD8^+^ T cells (Figure 7D) increased during acute infection, more in the apixaban-treated RMs than in the controls, but the fraction of Ki-67^+^ CD8^+^ T cells returned to baseline in both groups with ART. Altogether, these results indicate that apixaban had no effect on T cell recovery and proliferation in the gut.

### Apixaban has no discernible effect on monocyte subsets

**Figure 8:**
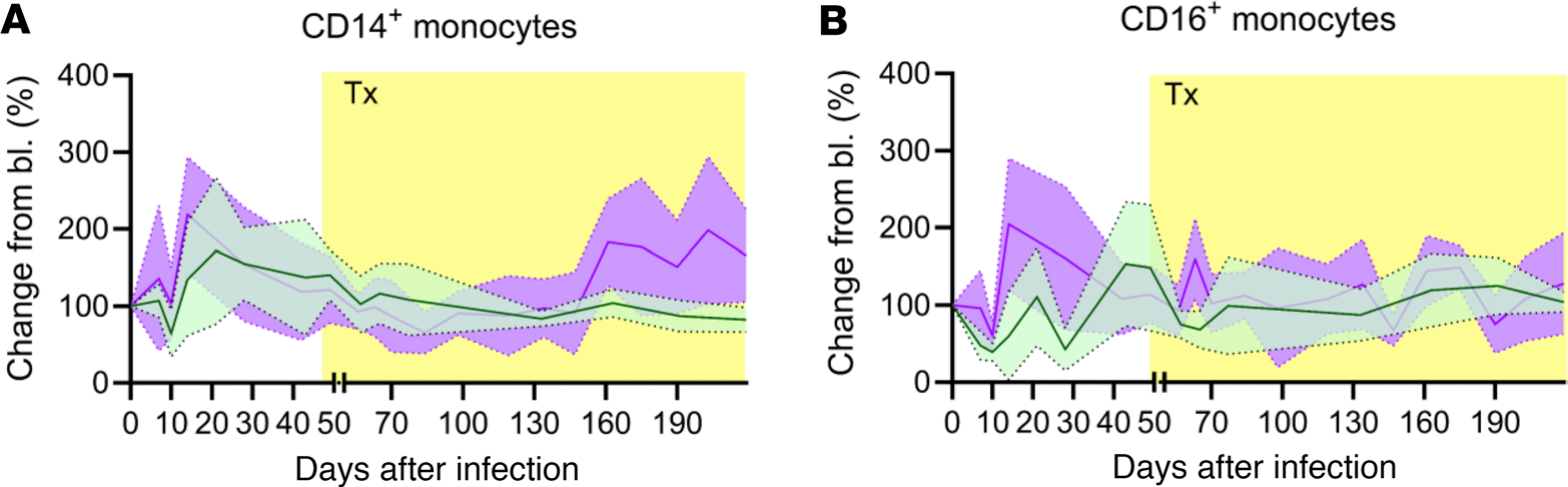
Apixaban administration does not alter the dynamics of monocyte populations in SIVmac-infected rhesus macaques receiving ART. The dynamics of the monocytes expressing either CD14 (**A**) or CD16 (**B**) were similar in the 2 groups. All the changes in these populations are illustrated as change from the baseline level (bl; %). Apixaban-treated RMs are shown in violet; controls are depicted in light green. Data are shown as mean ± SEM. Tx refers to both antiretrovirals and apixaban in the treated group.

CD14^+^ (Figure 8A) and CD16^+^ (Figure 8B) absolute counts were assessed using TruCounts. Upon the SIV infection, CD14^+^ monocytes increased in both groups, slightly more in the apixaban group. In controls, CD14^+^ monocytes dropped to half of their preinfection levels, before rapidly rebounding again to almost twice their baseline values. In the apixaban-treated RMs, CD14^+^ monocytes did not drop below the preinfection levels. The CD14^+^ monocytes then rapidly increased to slightly greater levels than in controls, followed by a steady decline throughout the follow-up period in both groups; CD14^+^ monocytes then started to increase in the apixaban group from 150 dpi on.

CD16^+^ monocytes remained relatively stable in both groups, with the exception of an acute depletion. Similar to CD14^+^ monocytes, they recovered better in the apixaban group than in controls. During chronic SIV infection, CD16^+^ monocyte levels were similar between the 2 groups.

Lack of significant differences between treated and untreated RMs for the dynamics of CD14^+^ and CD16^+^ monocytes, particularly after ART and apixaban initiation, led us to conclude that apixaban did not have any effect on monocytes.

### Apixaban has no effect on macrophage activation, inflammation, or the coagulation markers assessed in our study

**Figure 9:**
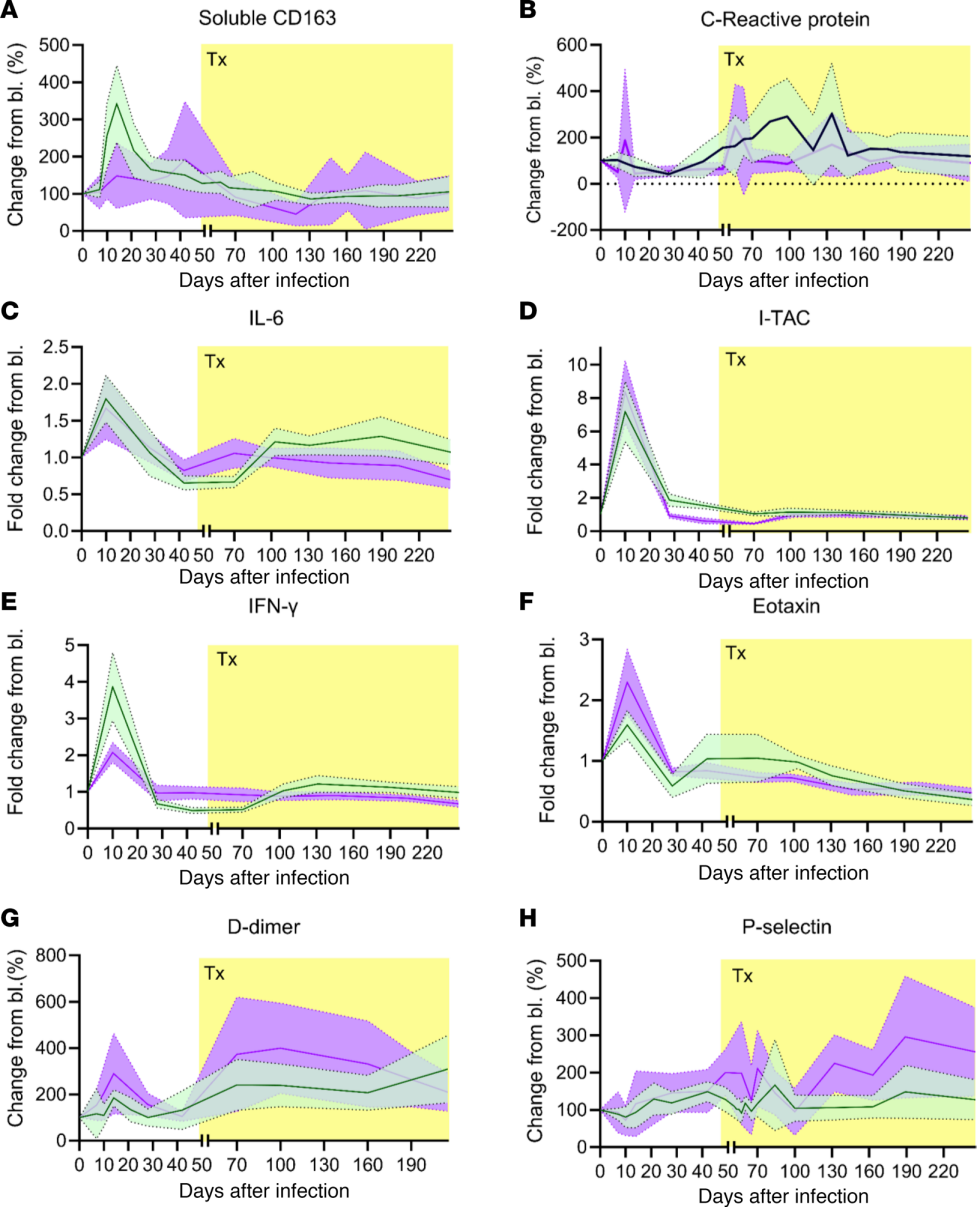
Apixaban administration does not alter the systemic inflammation and the coagulation status in SIVmac-infected rhesus macaques receiving ART. The dynamics of plasma levels of the biomarker of macrophage activation sCD163 (**A**), the acute phase reactant C-reactive protein (CRP) (**B**), the inflammatory mediators IL-6 (**C**), I-TAC (**D**), IFN-γ (**E**), and eotaxin (CCL-11) (**F**) were similar in the 2 groups, as were the dynamics of the hypercoagulation biomarker D-dimer (**G**) and endothelial/platelet activation marker P-selectin (**H**). All the changes in the levels of these biomarkers are illustrated as change from the baseline levels (% in **A**, **B**, **G**, and **H**; fold increase in **C**–**F**). Apixaban-treated RMs are shown in violet; controls are depicted in light green. Data are shown as mean ± SEM. Tx refers to both antiretrovirals and apixaban in the treated group; Bl, baseline.

Macrophage activation was assessed by monitoring soluble CD163 (sCD163; Figure 9A), which peaked for both groups during the acute infection, with the control group displaying a larger increase. Plasma sCD163 levels then decreased in the control group, while slightly increasing in the apixaban group. After the treatment initiation, sCD163 declined steadily in both groups, returning to baseline levels (Figure 9A).

Although studies have reported that anticoagulants reduce systemic inflammation, we did not observe significant differences in the levels of inflammation markers. Thus, C-reactive protein (CRP) levels steadily increased around 28 dpi, and they remained slightly increased in both groups after the apixaban and ART initiation (Figure 9B). There were no significant differences between the 2 groups with regard to the levels of inflammatory cytokines and chemokines, such as IL-6 (Figure 9C), I-TAC (Figure 9D), IFN-γ (Figure 9E), or eotaxin (Figure 9F).

Finally, coagulation status was assessed by monitoring D-dimer (Figure 9G), and endothelial/platelet activation was assessed by measuring P-selectin (Figure 9H). Although D-dimer levels were slightly higher in the treatment group during the acute infection and after treatment initiation, these differences did not reach significance (Figure 9G). In neither group was there a complete normalization of D-dimer levels after ART and/or apixaban. P-selectin was also similar in the 2 groups, with levels slightly elevated in the treatment group toward the end of the study (Figure 9H).

The similar dynamics of the biomarkers of inflammation and hypercoagulation in the 2 groups strongly indicate that apixaban had no effect on macrophage activation, inflammation, or coagulation status (based on the parameters that we measured here).

## Discussion

HIV/SIV infections are associated with a hypercoagulable state characterized by elevated plasma levels of biomarkers for coagulation (D-dimer) and inflammation (IL-6, sCD14, and CRP) (1–3, 52, 53). These biomarkers were shown to independently predict disease progression, non-AIDS comorbidities, and death (1, 54), even in individuals with HIV receiving ART (52). Furthermore, multiple studies have shown that, while ART can successfully suppress viral replication and reduce coagulation and inflammation biomarkers, their levels remain higher than in uninfected individuals (53).

We have established the pathogenic role of hypercoagulation in SIV-infected macaques (55) and shown that macaques are excellent models for the study of hypercoagulation and comorbidities in HIV infection, as they allow us to control for key variables of infection, such as time and route of SIV challenge, time of ART initiation, compliance with ART, and behavioral risk factors (40, 56–58). We also reported that such changes are characteristic of exclusively pathogenic infections; in the nonpathogenic infection of African green monkeys, no significant change in the functional activity of coagulation factors was observed upon SIV infection (40). Studies have also reported that alterations in the production and functional activity of coagulation factors occur during chronic SIV infection (59). Monocytes are an important source of coagulation factors (i.e., the tissue factor, the main initiator of the extrinsic coagulation cascade) (60). Monocytes have also been characterized as the immune cell subset involved in cardiovascular disease in both individuals with and without HIV (52, 61–63). The monocyte subsets diverge dramatically between pathogenic and nonpathogenic SIV infections, with SIV progression being related to systemic coagulopathy and increased capacity of monocytes to produce tissue factor upon activation (40).

In addition to their contribution to hypercoagulation through the production of cytokines and chemokines, monocytes are also central to the chronic inflammation observed during HIV/SIV infection, and it was proposed by us and others that the same monocyte subset expressing tissue factor upon activation may also be therefore implicated in persistent inflammation (40).

We previously administered a tissue-factor inhibitor (ixolaris) to reduce hypercoagulation and inflammation in young chronic SIV-infected pigtailed macaques. Ixolaris decreased inflammation and hypercoagulation, as illustrated by the plasma levels of IL-17 and D-dimer, yet was not associated with significant changes in CD4^+^ T cell counts or SIV viremia (40). Not only did ixolaris have an effect on inflammation, it also partially reduced T cell activation (40). As such, administration of a tissue-factor inhibitor confirmed our hypothesis that targeting hypercoagulation may help control chronic inflammation in patients with HIV. These previous results prompted us to pursue further studies, with the goal of identifying an effective treatment with minimal side effects that can be used in patients with HIV to counter hypercoagulation and systemic inflammation. Because ixolaris is an experimental drug that is not approved for usage in humans, we wanted to use an FDA-approved drug that has similar characteristics. We also sought to identify an anticoagulant that closely resembled our initial approach of blocking the coagulation cascade as early as possible, thereby limiting the feedback connections that rapidly occur once the coagulation process is initiated. We therefore selected apixaban, an FDA-approved factor Xa inhibitor used in clinical settings to prevent stroke and blood clots in patients with heart rhythm problems (e.g., nonvalvular atrial fibrillation) (46, 64). Apixaban fulfilled our selection criterion, as it acts high in the coagulation cascade, close to the site of action of the tissue factor (21). Finally, based on the analyses of the enzymes responsible for its metabolism, apixaban had a low probability to negatively interact with ART, which is indispensable in people with HIV. Indeed, a very recent study confirmed our rationale and showed that apixaban has a safer profile in patients with HIV and atrial fibrillation under ART (46).

Here, apixaban was administered to old RMs. The rationale was that the majority of people with HIV receiving ART worldwide are over the age of 50. Furthermore, even younger people with HIV show signs of accelerated aging (8), which triggers multiple comorbidities. Finally, the general population is aging, being affected by hypercoagulation, and experiencing more thrombotic events even in the absence of HIV. Altogether these arguments justified the selection of older NHPs for apixaban administration. We also reasoned that a successful anticoagulant therapy in older animals would be indicative of an even better response in young people. We also reasoned that we have better chances to affect coagulation, inflammation, and immune activation in an HIV/SIV system if we start the anticoagulant as early as possible. A more delayed start of apixaban would also have to deal with the metabolic effects of ART, which can increase the cardiovascular risk per se. Our goal was thus to counterbalance the negative impact of SIV and ART with our anticoagulant approach.

Apixaban administration to older SIV-infected RMs receiving ART had no discernible effect on SIV pathogenesis. None of the biomarkers associated with SIV disease progression were substantially modified by apixaban. Although the drug caused no major histological changes in liver or kidney or on the blood chemistry tests that would have been suggestive of liver or kidney dysfunction, slight increases in the levels of ALP, creatinine, and creatine kinase after apixaban administration indicate a need for a careful monitoring of apixaban in people with HIV receiving long-term treatment, even if it does not cause overt acute liver or kidney toxicity.

Surprisingly, apixaban did not normalize D-dimer in the old SIV-infected RMs. There are several potential reasons for this finding. First, we used a relatively low dose of apixaban, which we chose in agreement with clinical guidelines for older individuals with a low weight and increased hemorrhagic risk (65). Second, it is possible that edoxaban is more potent than apixaban. Previous studies showed that, in older populations, edoxaban is associated with higher risks of major bleeding, suggesting that it is more effective in reducing coagulability (66). Finally, in the human trial that used edoxaban, D-dimer and IL-6 were only modestly elevated. It is thus possible that the anticoagulants are more effective in individuals with moderate increases of D-dimer but not in those with an elevated hypercoagulable state. Actually, the authors state that their study could not assess the antiinflammatory effects of edoxaban among patients with high levels of inflammation and coagulation (41). We addressed these aspects by including older animals, which allowed us to show that in SIV-infected animals with high levels of D-dimer and inflammation, apixaban did not improve any of these parameters.

In spite of the persistent high levels of D-dimer, RMs receiving apixaban experienced bleeding complications that required interruption of apixaban administration throughout the follow-up period. The access to tissues collected at the necropsy allowed us to thoroughly assess the impact of the apixaban treatment at tissue sites. The pathological examination confirmed the presence of small hemorrhages in multiple RMs in multiple tissues, such as lung and pericardium. Together with a recurrent detection of hematuria in multiple animals, these results demonstrate that the apixaban dose could have not been increased without risks and justify our decision to end the study earlier due to lack of efficacy and occurrence of adverse effects.

Given apixaban’s lack of effect on immune activation, CD4^+^ T cell restoration, inflammation, and coagulation, along with increased risk of hemorrhages, we concluded that factor Xa inhibition does not appear to be a reasonable option to reduce the incidence of cardiovascular events in aging people living with HIV. The findings of a recent myocardial infarction and of an aortic dissection in apixaban-treated RMs support this conclusion. In people living with HIV, anticoagulant therapies should provide a beneficial impact on HIV pathogenesis by alleviating hypercoagulation, inflammation, and T cell activation.

Our results also strongly suggest that, if anticoagulants are investigated in the future as support therapy in people living with HIV, they should likely target different pathways and that, for such studies, the animal models are instrumental for both elucidating the poorly understood relationship between coagulation and inflammation and for testing the efficacy and safety of such therapies before they are implemented in people living with HIV.

## Methods

### Sex as a biological variable.

Only female RMs were included in this study. Sex cannot be studied as a biological variable in such small groups of NHPs.

### Ethics statement and animal care.

RMs were housed and maintained at the University of Pittsburgh Plum Borough animal facility, according to the standards of the Association for Assessment and Accreditation of Laboratory Animal Care (AAALAC) International, and experiments were approved by the University of Pittsburgh IACUC, protocol 18052719. The animals were cared for in compliance with the *Guide for the Care and Use of Laboratory Animals* (National Academies Press, 2011) (67). Animal procedures met or exceeded all standards of the Public Health Service’s Policy on the Humane Care and Use of Laboratory Animals (68). The animals were fed and housed according to regulations set forth by the *Guide for the Care and Use of Laboratory Animals* (National Academies Press, 2011) and the Animal Welfare Act (67).

All RMs included in this study were socially housed (paired) in stainless steel cages, had a 12-hour light/12-hour dark cycle, were fed twice daily with regular chow (Monkey Diet 5038, LabDiet), and received water ad libitum. A variety of environmental enrichment strategies were employed. Furthermore, RMs were observed twice daily, and any signs of disease or discomfort were reported to veterinarians for evaluation. For sample collection, RMs were anesthetized with 10 mg/kg ketamine HCl (Parke-Davis) or 0.7 mg/kg tiletamine HCl and zolazepam (Telazol, Fort Dodge Animal Health) injected intramuscularly. At the study completion, the animals were euthanized in accordance with the recommendations of the American Veterinary Medical Association (AVMA) Guidelines for the Euthanasia of Animals.

### Animal model, infections, and treatments.

Twelve RMs (*Macaca mulatta*), 17- to 21-year-old females, were included in this study. All macaques were i.v.-infected with 100 TCID_50_ of SIVmac239 as previously described (49) and were closely monitored during all stages of the study. Coformulated ART (5.1 mg/kg tenofovir, 50 mg/kg emtricitabine, and 2.5 mg/kg dolutegravir) (50) was initiated at 49 dpi in all RMs and maintained throughout the follow-up period, as described (51). Apixaban was also initiated (2.5 mg, orally, twice a day) at 49 dpi in 6 RMs to assess its impact on key parameters of SIV infection (survival, viral replication, CD4^+^ T cell counts and activation status, systemic inflammation, and coagulation status). The apixaban dose used in this study in RMs (old and weighing <10 kg) is the same as the one used for older people with low weight (<60 kg).

### Samples.

Blood was collected from all RMs twice prior to infection to establish the baselines (by calculating the averages of these 2 time points), approximately weekly after infection during the first 10 weeks, and then biweekly until the end of the follow-up period. Intestinal biopsies were collected prior to infection to establish baseline values for the parameters monitored during infection and treatment (69, 70). During acute infection, intestinal biopsies were taken at 10, 28, and 42 dpi. During chronic infection, intestinal biopsies were taken every month. Additional intestinal samples were collected during the necropsy.

Within 1 hour of blood collection, plasma was separated from whole blood by centrifugation (1,500*g* for 20 min), and PBMCs were isolated using Ficoll density gradient centrifugation and lymphocyte separation media (MP Biomedicals).

Intestinal biopsies were processed as described (71, 72). Briefly, they were washed with EDTA for 20 minutes at 37°C, then subjected to collagenase digestion at the same temperature for 40 minutes, both with agitation. The cell suspension was filtered and layered in a tube containing 2 mL of a 35% Percoll solution on top of 2 mL of a 60% Percoll solution. After centrifugation at 1500*g* for 20 minutes, lymphocytes were retrieved from between the 2 Percoll solutions. Freshly isolated cells were then used for flow cytometry.

### Plasma virus load quantification.

This step was performed as described (73). Briefly, viral RNA was extracted from plasma using Invitrogen PureLink Viral RNA/DNA mini kit (Life Technologies Corporation). Plasma RNA quantification was done using real-time PCR assays specific for SIVmac239. The primer and probe sequences amplify a conserved region of Gag and are as follows: SIVmac251F: 5′-GTC TGC GTC ATC TGG TGC ATT C-3′; SIVmac251R: 5′-CAC TAG GTG TCT CTG CAC TAT CTG TTT TG-3′; SIVmac251Probe: 5′- /56-FAM/CTT CCT CAG /ZEN/TGT GTT TCA CTT TCT CTT CTG CG/3IABkFQ/ -3′. Real-time PCR was performed utilizing an ABI QuantStudio 6 Pro machine (Applied Biosystems) with the following parameters: 95°C for 10 minutes, 45 cycles of 95°C for 15 seconds, 60°C for 1 minute. Plasma samples from the apixaban-treated RMs were also tested in the Quantitative Molecular Diagnostics Core within the AIDS and Cancer Virus Program of the National Cancer Institute.

### Antibodies and flow cytometry.

WBCs from blood and mononuclear cells isolated from intestinal biopsies and lymph nodes were immunophenotyped by flow cytometry, as described (70, 74). First, TruCount staining was performed on 50 μL of whole blood using CD45 (PerCP-Cy5.5, 3 μL, clone D058-1283), CD3 (V450, 2 μL, SP34-2), CD14 (PE-Cy7, 4 μL, M5E2), and CD16 (APC-Cy7, 4 μL, 3G8) antibodies (BD Biosciences). This allowed us to precisely quantify absolute numbers of circulating CD45^+^ cells, T cells, and monocytes. The exact counts of CD4^+^ and CD8^+^ T cells were determined by multiplying the exact number of CD3^+^ T cells by the percentage of CD4^+^ or CD8^+^ cells among CD3^+^ T cells that were determined in a different staining. CD14 and CD16 were used to identify circulating monocyte subsets. Whole peripheral blood (100 μL) and cells isolated from tissues were stained with fluorescently-labeled antibodies (all purchased from BD Biosciences, unless noted otherwise): CD3 (V450, 2 μL, clone SP34-2), CD4 (APC, 3 μL, L200), CD8 (PE-CF594, 3 μL, RPA-T8), CD28 (PE-Cy7, 3 μL, CD28.2), CD38 (FITC, 15 μL, AT-1) (STEMCELL Technologies), CD69 (APC-H7, 5 μL, FN50), CD95 (FITC, 15 μL, DX2), HLA-DR (PE-Cy7, 3 μL, L243), Ki-67 (FITC or PE, 20 μL, B56), and Live/Dead Aqua (200 μL of a 1:500 dilution, Thermo Fisher Scientific). For Ki-67 staining, cells were fixed, permeabilized with 1× BD Fix/Perm, then stained for Ki-67. All cells were washed, then fixed with BD Fix before being analyzed. Flow cytometry acquisitions were performed on an LSR II or LSR Fortessa flow cytometer (BD Biosciences). Flow data were analyzed using FlowJo (v10.8.0). Data were analyzed using FlowJo (v10.7.1), GraphPad Prism (v9.3.1), and SPICE 6. The gating strategy is presented in Supplemental Figure 1. Gating was first done on lymphocytes, then on CD3-positive T cells. Either CD4^+^ or CD8^+^ T cells were then selected, and expression of CD69, CD25, Ki-67, and HLA-DR and CD38 were then assessed on each of these populations (Supplemental Figure 1). Data were only included for analysis if a robust number of cells was present in the target population.

### Monitoring of inflammation using ELISA and Luminex.

Different soluble markers were quantified in the plasma of RMs using immunoassays: sCD163 (Macro163, IQ Products) and CRP (Monkey CRP ELISA kit, Life Diagnostics). Expression of cytokines and chemokines was quantified using a magnetic bead-based assay, the Cytokine 29-plex Monkey Panel (Thermo Fisher Scientific), and plates were read on a MAGPIX instrument. All ELISA and Luminex assays were performed according to the manufacturer’s instructions. Because of the relatively small sample size and the relatively large interindividual variation for some analytes, the levels of all markers are expressed as a fold-change compared with preinfection levels that were determined on 2–3 preinfection samples for all RMs.

### Endothelial activation.

We measured the plasma levels of P-selectin, a cell adhesion molecule on the surfaces of activated endothelial cells and platelets that acts as a receptor to support binding of leukocytes to activated platelets and endothelium. Plasma P-selectin levels are markedly elevated in inflammation and thrombogenic conditions (75). The plasma soluble P-selectin levels were measured with monkey P-selectin Platinum ELISA (eBioscience, Inc.), as per the manufacturer’s instructions.

### Coagulation status.

Coagulation status was estimated by determining plasma levels of D-dimer. D-dimer is a fibrin degradation product, a small protein fragment present in the blood after a blood clot is degraded by fibrinolysis. D-dimer was measured using a STAR automated coagulation analyzer (Diagnostica Stago) and an immunoturbidimetric assay (Liatest D-DI, Diagnostica Stago). Our previous studies optimized this assay for use in African green monkeys (3).

### Chemistry panels.

Comprehensive chemistry panels were performed on undiluted serum of RMs by IDEXX.

### Histology.

Heart, aorta, kidneys, brain, intestine (duodenum, jejunum, ileum, colon), and lung collected at the necropsy were fixed in 10% buffered formalin and embedded in paraffin. Next, 4 μm paraffin sections were stained with H&E for routine histopathology diagnosis, focusing on hemorrhagic lesions. We also routinely checked for pathology similar to that seen in people living with HIV, including renal thrombotic microangiopathy, arteriopathy, myocardial hypertrophy and fibrosis, atherosclerosis, infarction, and myocarditis (76–84).

### Statistics.

No formal sample size determination was performed. The size of the groups was based on historical data of the same parameters and prior experience. No data were excluded from the analyses unless the number of cells was too low to allow accurate flow cytometry analyses or samples were of poor quality. All assays were performed on 12 RMs at all time points when samples were available. For some experiments, assays could not be performed for all animals due to the lack of samples or to an insufficient number of cells to perform flow cytometry. The investigators were not blinded to allocation during experiments and outcome assessment, as they were involved in cell separation and preparation of the sample dilutions for the tests. Comparison of a single postinfection time point value with preinfection for each parameter was done using Mann-Whitney *U* tests. All statistical analyses were performed using GraphPad Prism. A *P* value of less than 0.05 was considered statistically significant.

### Study approval.

All animal experiments were approved by the IACUC of the University of Pittsburgh (Pittsburgh, Pennsylvania; protocol 18052719).

### Data availability.

Biomarker levels, as well as values for all data points shown in graphs, are reported in the Supporting Data Values file.

## Author contributions

CX, HA, LC, VK, RS, SS, TH, KDR, JK, CB, NA-S, MD, SK, CA, and IP acquired and analyzed data and performed in-depth/comprehensive analysis of biomarker data. IP, CA, MJF, and IS designed research studies. LC, CA, IP, IS, and MJF wrote and reviewed the manuscript. CA and IP provided project management, insured funding, and coordinated the studies.

## Conflict of interest

The authors have declared that no conflict of interest exists.

## Funding support

This work is the result of NIH funding, in whole or in part, and is subject to the NIH Public Access Policy. Through acceptance of this federal funding, the NIH has been given a right to make the work publicly available in PubMed Central.

- NIH grants R01 HL117715 (to IP), R01 HL123096 (to IP), and R01 HL154862 (to IP and MJF) from the National Heart, Lung, and Blood Institute.
- NIH grants R01 DK119936 (to CA), R01 DK113919 (to IP and CA), R01 DK131476 (to CA), and R01 DK130481 (to IP) from the National Institute of Diabetes and Digestive and Kidney Diseases.
- NIH grants R01AI179317 (to IP) and R01 AI119346 (to CA) from the National Institute of Allergy and Infectious Diseases.
- Intramural Research Program of NIH (to IS).

## Supplementary Material

Supplemental data

Supporting data values

## Version 1 04/08/2026

Electronic publication
