# Supplementary material for: Impact of a factor Xa inhibitor (apixaban) on SIV pathogenesis and response to antiretroviral therapy
Source: JCI Insight. 2026 Apr 8;11(7):e202434. doi: 10.1172/jci.insight.202434 (PMC13134728; doi:10.1172/jci.insight.202434)
Supplement: Supplemental data [file jciinsight-11-202434-s163.pdf]

Impact of a Factor Xa Inhibitor (Apixaban) on SIV Pathogenesis and Response to Antiretroviral Therapy

----

Cuiling Xu,[1,2] Haritha Annapureddy,[1] Lilly Carson,[1] Vansh Khurana,[1] Ranjit Sivanandham,[1] Sindhuja Sivanandham,[1] Tianyu He,[1,2] Kevin D. Raehtz,[1] Janet Kim,[1] Christie Biber,[1] Norma Arbujas-Silva,[1] Mohammed Daira,[1] Sudhapriya Kandasamy,[1] Matthew Feinstein,[3,4] Irini Sereti#,[5] Cristian Apetrei[2,6] & Ivona Pandrea[1,6*]

SUPPLEMENTAL MATERIALS

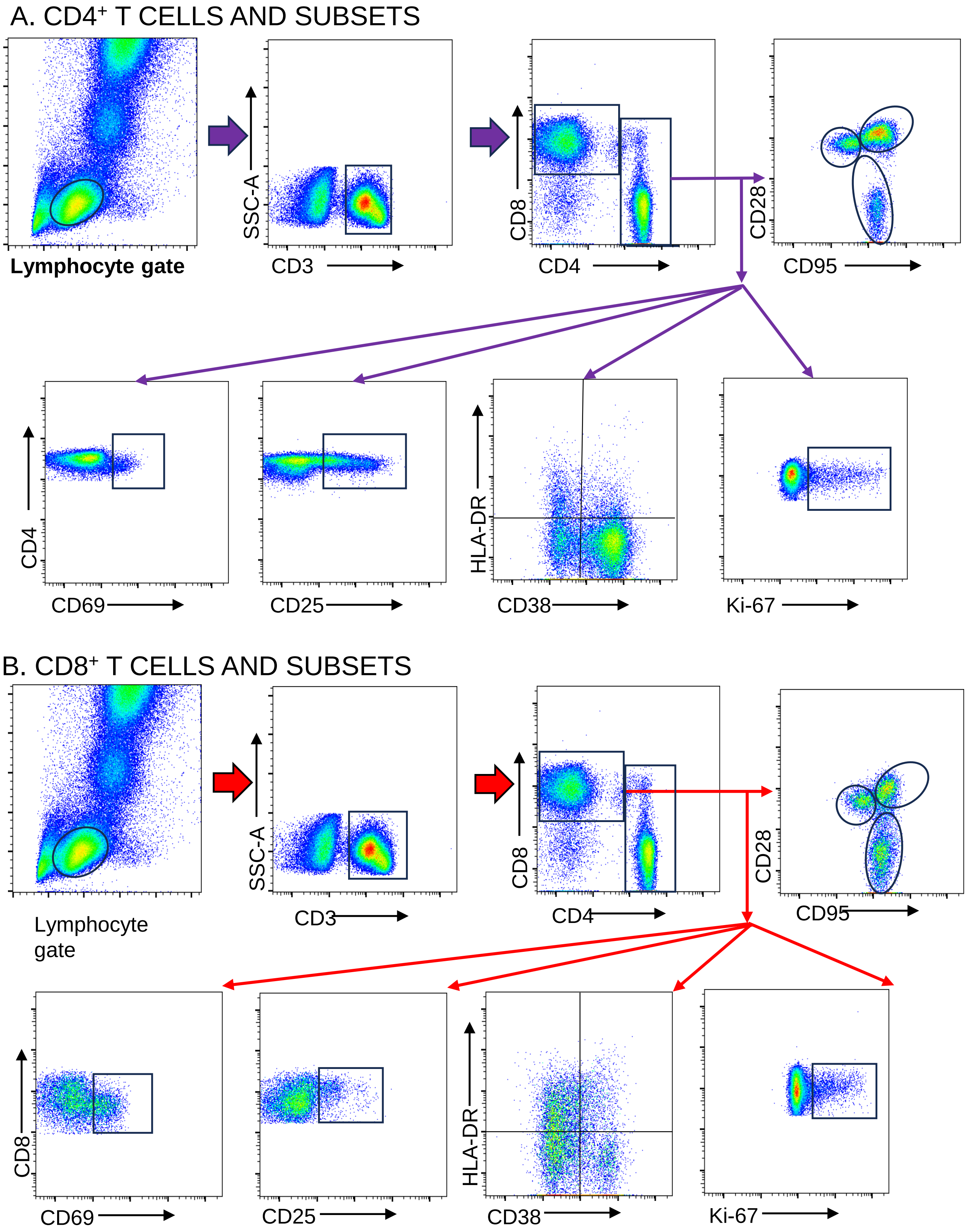

Supplemental Figure 1. Gating strategy employed to characterize $CD4^+$ T cells (A) and $CD8^+$ T cells, their subsets and their activation and proliferation status. $CD4^+$ and $CD8^+$ T cells were gated on singlets followed by lymphocytes and $CD3^+$; $CD4^+$ and $CD8^+$ T-cell expression of CD28 and CD95 was used characterize the memory subsets: naïve ($CD28^+$ $CD95^{neg}$); central memory (CM: $CD28^+$ $CD95^{neg}$) and effector memory ($CD28^{neg}$ $CD95^+$) (upper right panels in A and B); then, $CD4^+$ and $CD8^+$ T-cell activation and proliferation status was assessed based on the frequency of expression of CD69, CD25, CD38 and HLA-DR and Ki-67 by the $CD4^+$ (lower panels in A) and $CD8^+$ T cells (lower panels in B).

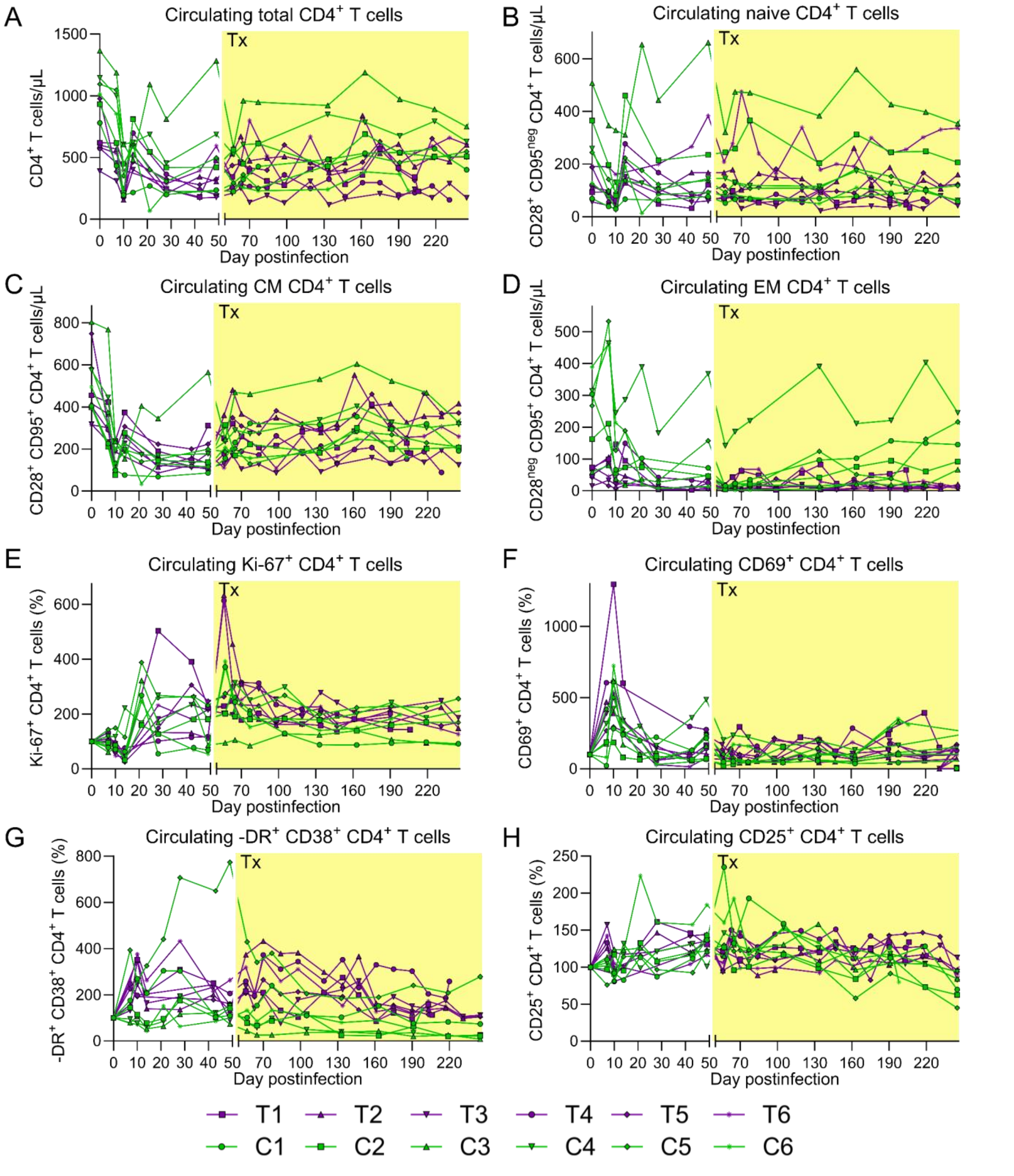

Supplemental Figure 2. Apixaban administration does not alter the dynamics of circulating CD4+ T cells in SIVmac-infected rhesus macaques (RMs) receiving antiretroviral therapy (ART). The magnitude and timing of the CD4+ T-cell changes were similar in the two groups with regard to total circulating CD4+ T cells (A), as well as the memory subtypes: naïve (B), central memory (CM) (C) and effector memory (EM) (D). Peripheral CD4+ T-cell activation and proliferation were not different between the two groups, as illustrated by the dynamics of the CD4+ T cells expressing CD69 (E), CD25 (F), CD38 and HLA-DR (G) and Ki-67 (H). The CD4+ T cells and their memory subsets (A-D) are shown as absolute counts/µL. All the changes in frequency of CD4+ T cells expressing activation markers (E-H) are illustrated as change from the baseline levels (%). Apixaban-treated RMs are depicted in violet, controls are depicted in light green. Tx refers to both antiretrovirals and Apixaban in the treated group; Bl-baseline.

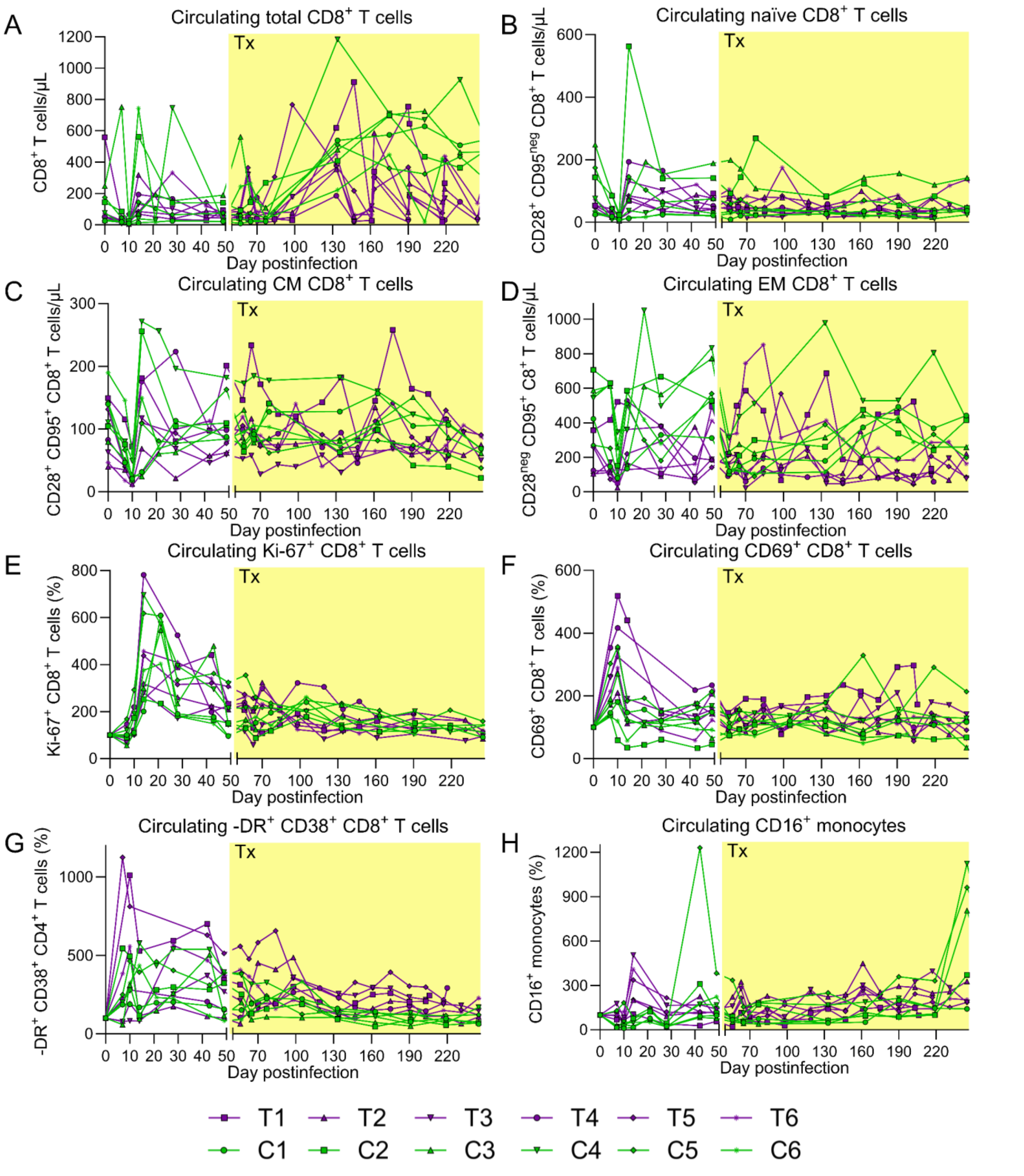

Supplemental Figure 3. Apixaban administration does not alter the dynamics of circulating CD8⁺ T cells in SIVmac-infected rhesus macaques (RMs) receiving antiretroviral therapy (ART). The dynamics of CD8⁺ T cells were similar in the two groups with regard to total circulating CD8⁺ T cells (A), as well as the memory subtypes: naïve (B), central memory (CM) (C) and effector memory (EM) (D). Peripheral CD8⁺ T-cell activation and proliferation were not different between the two groups, as illustrated by the dynamics of CD8⁺ T cells expressing CD69 (E), CD25 (F), CD38 and HLA-DR (G) and Ki-67 (H). The CD8⁺ T cells and their memory subsets (A-D) are shown as absolute counts/μL. All the changes in frequency of CD8⁺ T cells expressing activation markers (E-H) are illustrated as change from the baseline levels (%). Apixaban-treated RMs are depicted in violet, controls are depicted in light green. Tx refers to both antiretrovirals and Apixaban in the treated group; Bl-baseline.

Supplemental Table 1. Cardiovascular lesions in Apixaban-treated and control SIV-infected, old Rhesus macaques on ART

	Controls						Apixaban-treated					
Cardiovascular lesions	C1	C2	C3	C4	C5	C6	T1	T2	T3	T4	T5	T6
Infarction	Y (chr)			Y (chr)							Y (ac)	Y (chr)
Focal myocardial cell death/hypoxia			Y			Y			Y			Y
Fat infiltration	Y	Y	Y		Y			Y	Y			Y
Myocardial Inflammation	Y	Y		Y				Y				
Myocardial hypertrophy				Y		Y						Y
Myocardial fibrosis				Y								
Pericardial hemorrhage							Y			Y		
Atherosclerosis Coronary							Y					
Abdominal aorta dissection						Y	Y					
Thoracic aorta dissection							Y					

C-control; T-treated; Y-lesion present; chr-chronic; ac-acute

Supplemental Table 2. Semiquantitative assessment of the histopathologic lesions found in Apixaban-treated and control SIV-infected, old rhesus macaques on ART

H&E	CONTROLS							APIXABAN-TREATED						
	C1	C2	C3	C4	C5	C6	Mean	T1	T2	T3	T4	T5	T6	Mean
Thoracic Aorta														
Atherosclerosis														
Aorta (plaque)	1	1	2	2	0	1	1.2	1.5	1.5	1	0	0	1	0.8
Abdominal Aorta														
Atherosclerosis														
Aorta (plaque)	1	0.5	3	8	2	5	3.25	5	2.5	3	6	2	1	3.25
Lung														
Stasis	9	1	9	5	3	1	4.7	8	5	4	7	5	1	5
Transudate	2	0	0	0	0	0	0.3	0	2	0	0	0	0	0.3
Inflammation	1	2	3	3	1	1	1.8	1	1	2	1	1	1	1.2
Hemorrhage	0	0	0	0	0	0	0	2	2	0	0	1	0	0.8
Liver														
Steatosis	6	9	4	3	3	2	4.5	3	1.5	3	1	2	6	2.75
Inflamation	1	1	2	1	1	2	1.3	2	2	4	1	1	3	2
Cholestasis	0	0	1	0	0	0	0.2	0	1	0	0	0	0	0.2
Kidney														
Hemorrhage	0	0	0	0	0	0	0	0	0	0	1	0	1	0.3
Interstitial infl.	2	1	2	1	1	1	1.3	3	2	2	1	1	2	1.8
Glomerulonephritis	4	3	4	3	3	2	3.2	1	4	4	1	2	2	2.3
Stasis	3	1	3	1	3	1	2	1	5	3	1	3	9	3.6
Hyaline cast	1	4	1	2	3	3	2.3	4	2	4	0	1	0	1.8
Amyloidosis	0	0	0	0	0	0	0	0	0	1	0	0	0	0.2
SLN														
Lymphoid hyperpl.	3	4	N/A	6	0	3	3.2	0	3	3	0	4	0	1.7
Lymphoid depletion	3	8	N/A	1	6	4	4.4	7	3	4	7	2	9	5.3
Fat infiltration	8	1	N/A	0	3	5	3.4	0	5	5	3	0	2	2.5
Amyloidosis/Hyaline	3	0	N/A	0	2	5	2	0	0	0	2	0	0	0.3
MLN														
Lymphoid hyperpl.	2	2	0	0	N/A	N/A	1	0	0	0	0	3	3	1
Lymphoid depletion	5	3	8	8	N/A	N/A	6	9	8	9	9	2	2	6.5
Fat infiltiration	2	0	0	0	N/A	N/A	0.5	2	0	0	1	0	0	0.5
Amyloidosis/Hyaline	5	2	3	2	N/A	N/A	3	2	3	4	5	1	2	2.8

Severity of lesions ranges between 0 (absent) to 9 (the most severe)
